# When a Slice Is Not Enough! Comparison of Whole-Brain versus Standard Limited-Slice Perfusion Computed Tomography in Patients with Severe Traumatic Brain Injury

**DOI:** 10.3390/jcm8050701

**Published:** 2019-05-17

**Authors:** Shannon Cooper, Cino Bendinelli, Andrew Bivard, Mark Parsons, Zsolt J. Balogh

**Affiliations:** 1 Department of Traumatology, John Hunter Hospital, Newcastle 2300, Australia; shannon.cooper02@gmail.com (S.C.); cino.bendinelli@hnehealth.nsw.gov.au (C.B.); 2 Faculty of Medicine, University of Newcastle, Newcastle 2300, Australia; 3 Department of Neurology, Royal Melbourne Hospital, Victoria 3050, Australia; abivard@unimelb.edu.au (A.B.); Mark.Parsons@mh.org.au (M.P.); 4 Faculty of Medicine, University of Melbourne, Melbourne 3050, Australia

**Keywords:** severe traumatic brain injury, perfusion computed tomography, PCT

## Abstract

Introduction: Cerebral perfusion computed tomography (PCT) provides crucial information in acute stroke and has an increasing role in traumatic brain injury (TBI) management. Most studies on TBI patients utilize 64-slice scanners, which are limited to four brain slices (limited-brain PCT, LBPCT). Newer 320-slice scanners depict the whole brain perfusion status (WBPCT). We aimed to identify the additional information gained with WBPCT when compared to LBPCT. Patients and methods: Forty-nine patients with severe TBI were investigated within 48 h from admission with WBPCT. Findings from LBPCT were compared with findings from WBPCT. Results: A perfusion abnormality was identified in 39 (80%) and 37 (76%) patients by WBPCT and LBPCT, respectively (*p* = 0.8). There were 90 and 68 perfusion abnormalities identified by WBPCT and LBPCT, respectively (*p* < 0.001). In the 39 patients with a perfusion abnormality detected by WBPCT, 15 (38%) had further perfusion abnormalities outside the LBPCT area of coverage. Thirty-six (92%) patients had a larger perfusion abnormality upon WBPCT compared with LBPCT. Additional information gained showed some statistically significant correlation with clinical outcome. Conclusions: In severe TBI patients, WBPCT provides extra information compared to LBPC. The limitations of LBPCT should be considered when evaluating studies reporting on PCT findings and their association with outcomes.

## 1. Introduction

Neuroimaging obtained with computed tomography (CT) plays a crucial role in the clinical management of patients with severe traumatic brain injury (sTBI) [1]. Cerebral perfusion computed tomography (PCT) is a logistically non-demanding imaging modality that can be obtained on widely available multidetector CT scanners [1,2]. PCT depicts cerebral perfusion and has an established role in the management of patients with acute stroke [3,4]. A limited number of studies exist that have investigated PCT in the context of sTBI. These have demonstrated and association of the PCT findings with cerebral autoregulation [5], cerebral pressure [6], and functional outcome [7,8]. Most of the reported experience with PCT in sTBI patients [5,6,8,9,10] rely on 64-slice CT scanners which are able to generate up to four single axial slices 5 mm apart giving a z-axis coverage of about 2 cm. Newer 320slice CT scanners can provide 16 cm of z-axis coverage and are able to image the whole brain in a single rotation. Studies on stroke patients have demonstrated the superiority of 320-slice whole-brain PCT (WBPCT) when compared to the limited-brain PCT (LBPCT) in terms of additional diagnostic details [11,12]. 

No studies have yet compared LBPCT with WBPCT in patients with sTBI. In these patients, multiple lesions (typically not restricted to vascular territories) are the norm, and thus the increased field of coverage would be expected to detect more lesions. We hypothesized that in sTBI patients, WBPCT would provide extra diagnostic information when compared to LBPCT.

## 2. Patients and Methods

All subjects gave their informed consent for inclusion before they participated in the study. The study was conducted in accordance with the Declaration of Helsinki, and the protocol was approved by the Hunter New England Ethics Committee (11/12/14/4.03).

This study was undertaken at a Level 1 trauma centre. All patients investigated with WBPCT for sTBI were identified and retrospectively recruited in this study. Indication for WBPCT was an sTBI and not improving neurology at 48 h from admission despite proactive medical and surgical treatment. 

Data collected included age, gender, mechanism of injury, Glasgow Coma Scale prior to intubation, injury severity score (ISS), head and neck abbreviated injury score (HNAIS), venous lactate and base deficit on arrival to the emergency department, mortality and length of stay in intensive care unit (ICU). Simultaneous non-contrast CT findings were scored using the Rotterdam CT classification. Long-term functional outcome data were evaluated at six months using the Glasgow Outcome Scale Extended (GOSE), which was then dichotomised into favourable outcome (GOSE 5–8) and unfavourable outcome (GOSE 1–4).

To simulate an LBPCT, perfusion maps of four continuous 5 mm axial slices were selected beginning immediately above the orbits at the level of the foramen of Monro. This resulted in a 2 cm area of analysis covering a similar region to earlier studies [5,6,8,9,10]. These were scored in a binary fashion as having a visible area of perfusion abnormality or no visible perfusion abnormality. In addition, the number of discrete areas of perfusion abnormality detected was evaluated. The WBPCT images were reviewed and scored in a similar fashion. In patients with a perfusion abnormality on LBPCT, three other binary variables were scored. The first was whether there were any new areas of perfusion abnormality seen in the WBCT images that were not visible in the LBPCT images. The second variable was whether there was an increase in the axial dimension visible by WBPCT of any area of perfusion abnormality that was already visible by LBPCT. The third variable was whether there was an increase in the longitudinal dimension visible by WBPCT of any area of perfusion abnormality already visible by LBPCT. All WBPCT and LBPCT images were reviewed and scored by a consultant stroke neurologist (AB).

The number of areas of perfusion abnormality detected by WBPCT and LBPCT were compared. The sensitivity, specificity positive predictive value and negative predictive value for detecting any perfusion abnormality by LBPCT compared to WBPCT was calculated. As a secondary comparison, the clinical features and outcomes of patients who had a new area of perfusion abnormality detected by WBPCT were compared with those of patients without new detected abnormalities. A similar comparison was performed for those patients with an increase in the axial dimension of an area of perfusion abnormality visible by LBPCT.

All statistical analyses were carried out using IBM SPSS version 24. Continuous parametric data are presented as a mean and standard deviation and were compared using Student’s t-test. Continuous non-parametric data are presented with a median and interquartile range (IQR) and were compared using a Wilcoxin sign-rank test for paired observations or a Mann–Whitney U test for unpaired observations. Dichotomous and categorical data are presented as a percentage over the total number of patients observed and were compared using Fisher’s exact test. For all comparisons, a *p* value of less than 0.05 was considered significant. 

## 3. Results

Forty-nine patients were identified and included in the study. Their demographic and clinical findings are shown in Table 1.

This was a cohort of severely injured trauma patients (mean ISS: 30) with severe sTBI (median HNAIS: 4), requiring prolonged ICU stay (median days: 10), burdened by high mortality (16%) and poor functional outcome (unfavourable in 59%). 

A perfusion abnormality was identified in 39 (80%) and 37 (76%) patients, by WBPCT and LBPCT, respectively (*p* = 0.8). LBPCT missed perfusion deficits in the superior frontal lobe and inferior temporal lobe. LBPCT had a sensitivity of 95%, a specificity of 100%, positive predictive value 100% and negative predictive value of 83% in detecting any perfusion abnormality, when referenced to WBPCT. LBPCT detected a total of 68 (median per patient: (1) lesions compared to a total of 90 (median per patient: (2) lesions detected by WBPCT (*p* < 0.001) (Table 2). 

**Figure 1:**
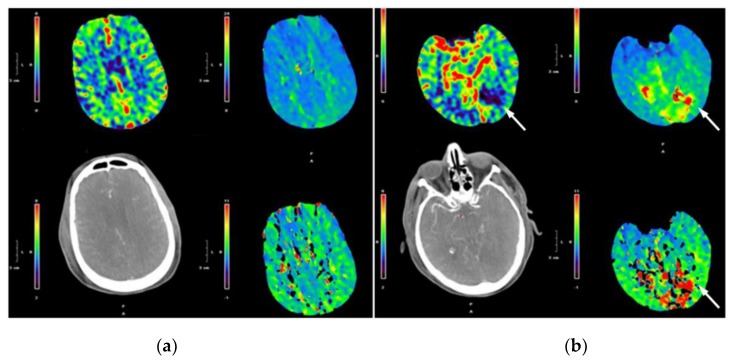
Example of LBPCT (**a**) and WBPCT (**b**) demonstrating a new area of perfusion abnormality not visible by LBPCT (arrowed).

In the 39 patients with a perfusion abnormality detected by WBPCT, 15 (38%) demonstrated a perfusion abnormality that was outside the area covered by LBPCT (Figure 1). 

In comparison with LBPCT results, the perfusion abnormality appeared larger in WBPCT images in 12 (32%) and 36 (92%) patients in the axial and longitudinal axis, respectively (Table 3).

When patients who presented additional abnormalities by WBPCT were compared with those who did not, there were no statistically significant differences demonstrated, apart from a higher lactate on arrival (Table 4 and Table 5).

## 4. Discussion

Optimizing cerebral perfusion is one of the main goals in the management of patients with sTBI [13]. Non-contrast cranial CT is the gold standard imaging modality in guiding treatment decisions but does not provide any direct information about cerebral perfusion [14]. Magnetic resonance, Xenon-CT and PCT can instead provide detailed cerebral perfusion maps [14]. PCT has the advantages of being logistically less demanding and being achievable with widely available multidetector CT scanners. Perfusion data are obtained by monitoring the first pass of intravenous contrast material through the cerebral vessels. Post-processing of the data allows the quantification of the perfusion parameters of regional cerebral blood flow, regional cerebral blood volume, and mean transit time [14].

This study analysed the diagnostic yield of PCT performed with two different technologies. LBPCT is obtained with 64-slice CT scanners, depicts up to four single axial slices (usually located at the level of the third ventricle) and covers up to 4 cm on the z-axis of the brain parenchyma. WBPCT is obtained with more advanced 320-slice CT scanners and images the whole brain (16 cm of z-axis coverage). The difference in z-axis coverage is particularly important when imaging sTBI patients, whose lesions are likely to conform to vectors of force rather than to vascular territories. As hypothesized, WBPCT provided additional information in almost all patients (97%): the areas of altered hypoperfusion were more frequent in almost 40%, larger in more than 30% and otherwise missed in 38% of patients. These results are comparable to the findings of similar studies performed in the stroke population which demonstrated a new lesion in 42% of patients when imaged with WBCT rather than LBPCT [11,12]. It is worth noting that in the stroke patients, only 14% had a new lesion demonstrated in a separate vascular territory by WBPCT. This differs from our findings, where almost all new perfusion abnormalities were in a different territory to the area of perfusion abnormality detected by LBPCT. This difference is in keeping with the different mechanisms of the pathology. 

PCT has a promising role in the management of patients with Severe TBI. The vast majority of published studies have utilized 64-slice scanners technology and obtained LBPCT [1,4,5,6,8,9,10]. According to the findings of this study, the greater diagnostic yield of WBPCT provides additional useful information. It is therefore highly likely that the current literature might be underestimating the role of PCT in patients with severe TBI.

This study also tried to assess whether the extra information gained by WBPCT might be clinically relevant. When patients with additional findings on WBPCT were compared with those without, the only statistically significant difference observed was a higher lactate level at admission. This might be explained by the correlation between poor global perfusion, typical of the severely injured trauma patient in shock, and poor overall brain perfusion, with subsequent larger brain perfusion deficits. Although other differences failed to reach statistical significance, patients with a larger axial dimension of perfusion abnormality upon WBPCT showed a trend towards worse six-month clinical outcome. This study is underpowered to explore this finding. On power analysis, a study group of 100 patients would have been necessary to reach statistically significant (and clinically relevant) differences.

This study has weaknesses that should be acknowledged. The PCT maps were stored as image files, which enabled them to be scored qualitatively but not to perform an accurate volumetric analysis [15]. A computerized volumetric analysis would allow the determination of exactly how much extra abnormally perfused or severely ischaemic tissue WBPCT detected compared with LBPCT.

It is important to underline that PCT in patients with sTBI remains a research tool. Its emerging role in outcome prognostication [7], cerebral contusion evaluation [9], and cerebral vascular autoregulation [5] requires further validation from lager studies before it can become standard of care.

## 5. Conclusions

Our study demonstrates that in the majority of sTBI patients, WBPCT provides additional crucial information when compared to LBPCT. WBPCT should be favoured in both the clinical and the research setting. Findings from studies that have used LBPCT should be interpreted cautiously.

## Figures and Tables

**Table 1:** Demographic, clinical and outcome details of the study population.

Number of Patients		49
	Age (years): median (IQR)	35 (19.5–50.5)
	Male: *n* (%)	42 (86%)
	Blunt trauma: *n* (%)	49 (100%)
Clinical variables		
	Pre-intubation GCS: median (IQR)	5 (3–6)
	ISS: mean (SD)	30 (9.7)
	HNAIS: median (IQR)	4 (3–5)
	Lactate on arrival: median (IQR)	2.8 (0.7–4.9)
	Base deficit on arrival: median (IQR)	2.4 (1.6–6.4)
	Rotterdam NCCT score: median (IQR)	2 (2–2)
Outcome variables		
	ICU length of stay (days): median (IQR)	10 (6–15)
	Mortality: *n* (%)	8 (16%)
	Unfavourable GOSE at 6 months: *n* (%)	39 (59%)

GCS: Glasgow coma scale; HNAIS: Head and neck abbreviated injury score; ICU Intensive care unit; IQR Interquartile range; NCCT non-contrast CT; SD Standard deviation; Unfavourable GOSE: Glasgow Outcome Scale Extended between 1 and 4.

**Table 2:** Comparison of WBPCT versus LBPCT findings.

PCT Result	LBPCT	WBPCT	*p*-Value
Perfusion abnormality: *n* (%)	37 (76)	39 (80)	0.8
Perfusion abnormalities detected: *n*	68	90	
Perfusion abnormalities detected per patient: median (IQR)	1 (1–2)	2 (1–3)	<0.001

PCT: perfusion computer tomography; LBPCT: limited-brain PCT; WBPCT: whole-brain PCT.

**Table 3:** Additional findings detected by WBPCT when compared with LBPCT in 39 patients with perfusion abnormality identified by WBPCT.

New Finding by WBPCT	*n* (%)
Additional perfusion abnormality	15 (38%)
Increased axial size	12 (31%)
Increased longitudinal size	36 (92%)
Any additional information on WBPCT	38 (97%)
New abnormality or increased axial size	23 (59%)

**Table 4:** Comparison of patients with a new perfusion abnormality detected by WBPCT versus those without new abnormalities.

		New Abnormality	No New Abnormality	*p*
Number of patients		15	34	
	Age (years): median (IQR)	35 (22.5–60.0)	34 (23.0–53.0)	0.672 ^a^
	Male: *n* (%)	13 (87%)	29 (85%)	1.000 ^b^
	Pre-intubation GCS: median (IQR)	5 (3.5–7.0)	4 (3.0–6.0)	0.679 ^b^
	ISS: mean (SD)	24.6 (9.2)	13.1 (8.8)	0.727 ^c^
	HNAIS: median (IQR)	4 (2–5)	4 (3–5)	0.458 ^a^
	Lactate on arrival: median (IQR)	3.0 (2.3–4.0)	2.6 (1.7–4.1)	0.291 ^a^
	Base deficit on arrival: median (IQR)	1.8 (1.0–3.9)	2.8 (1.3–5.4)	0.515 ^a^
	Rotterdam NCCT score: median (IQR)	2 (2.0–2.5)	2 (2.0–2.0)	0.946 ^a^
	ICU length of stay (days): median (IQR)	12 (8–16)	9 (6–15)	0.415 ^a^
	Mortality: *n* (%)	2 (13%)	6 (18%)	0.328 ^b^
	Favourable GOSE at 6 months: *n* (%)	6 (40%)	14 (41%)	0.597 ^b^

^a^ Mann–Whitney U test; ^b^ Fisher’s Exact test; ^c^ Student’s *t*-test.

**Table 5:** Comparison of patients with increased axial dimension of perfusion abnormality detected by WBPCT versus those without any increase in abnormality’s axial dimension.

		Increased Axial Dimension on WBPCT	No Increased Axial Dimension on WBPCT	*p*-Value
Number of patients		12	37	
	Age (years): median (IQR)	36 (23.0–50.0)	35 (23.0–55.0)	0.963 ^a^
	Male: *n* (%)	10 (83%)	32 (87%)	1.000 ^b^
	Pre-intubation GCS: median (IQR)	4 (3.0–6.5)	5 (3.0–6.0)	0.556 ^b^
	ISS: mean (SD)	33.1 (8.4)	30.0 (8.9)	0.265 ^c^
	HNAIS: median (IQR)	4 (3–5)	4.5 (2.5–5.5)	0.864 ^a^
	Lactate on arrival: median (IQR)	4.1 (3.3–5.1)	2.5 (1.9–3.7)	0.039 ^a^
	Base deficit on arrival: median (IQR)	2.3 (0.6–5.4)	2.4 (1.3–4.3)	0.780 ^a^
	Rotterdam NCCT score: median (IQR)	2 (2.0–3.5)	2 (1.5–2.0)	0.212 ^a^
	ICU length of stay (days): median (IQR)	13 (9–19)	9 (6–15)	0.108 ^a^
	Mortality: *n* (%)	3 (25%)	5 (13%)	0.385 ^b^
	Favourable GOSE at 6 months: *n* (%)	2 (17%)	18 (49%)	0.089 ^b^

^a^ Mann–Whitney U test; ^b^ Fisher’s Exact test; ^c^ Student’s *t*-test.

## References

[1] Douglas D.B., Muldermans J.L., Wintermark M. (2018). Neuroimaging of brain trauma. Curr. Opin. Neurol..

[2] Bendinelli C., Bivard A., Nebauer S., Parsons M.W., Balogh Z.J. (2013). Brain CT perfusion provides additional useful information in severe traumatic brain injury. Injury.

[3] Bivard A., Levi C., Krishnamurthy V., McElduff P., Miteff F., Spratt N.J., Bateman G., Donnan G., Davis S., Parsons M. (2015). Perfusion computed tomography to assist decision making for stroke thrombolysis. Brain.

[4] Wintermark M., Sesay M., Barbier E., Borbely K., Dilon W.P., Eastwood J.D., Glenn T.C., Grandin C.B., Pedraza S., Soustiel J.F. (2005). Comparative overview of brain perfusion imaging techniques. Stroke.

[5] Wintermark M., Chiolero R., Melle G.V., Revelly J.P., Porchet F., Regli L., Maeder P., Meuli R., Schnyder P. (2006). Cerebral vascular autoregulation assessed by perfusion-CT in severe head trauma patients. J. Neurorad..

[6] Wintermark M., Chiolero R., Melle G.v., Revelly J.P., Porchet F., Regali L., Meuli R., Schnyder P., Maeder P. (2004). Relationship between brain perfusion computed tomography variables and cerebral perfusion pressure in severe head trauma patients. Crit. Care Med..

[7] Bendinelli C., Cooper S., Evans T., Bivard A., Pacey D., Parsons M., Balogh Z.J. (2017). Perfusion abnormalities are frequently detected by early CT perfusion and predict unfavourable outcome following severe traumatic brain injury. World J. Surg..

[8] Wintermark M., Melle G.v., Schnyder P., Revelly J.-P., Porchet F., Regali L., Meuli R., Maeder P., Chioléro R. (2004). Admission perfusion CT: Prognostic value in patients with severe head trauma. Radiology.

[9] Soustiel J.F., Mahamid E., Goldsher D., Zaaroor M. (2008). Perfusion-CT for early assessment of traumatic cerebral contusions. Neuroradiology.

[10] Soustiel J.F., Mor N., Zaaroor M., Goldsher D. (2006). Cerebral perfusion computerized tomography: Influence of reference vessels, regions of interest and interobserver variability. Neuroradiology.

[11] Page M., Nandurkar D., Crossett M.P., Stuckey S.L., Lau K.P., Kenning N., Troupis J.M. (2010). Comparison of 4cm Z-axis and 16cm Z-axis multidetector CT perfusion. Eur. Rad..

[12] Dorn F., Muenzel D., Meier R., Poppert H., Rummeny E.J., Huber A. (2011). Brain perfusion CT for acute stroke using 256-slice CT: Improvement in diagnostic information by large volume coverage. Eur. Rad..

[13] Carney N., Totten A.M., O’Reilly C., Ullman J.S., Hawryluk G.W.J., Bell M.J., Bratton S.L., Chesnut R., Harris O.A., Kissoon N. (2017). Guidelines for the management of severe traumatic brain injury, Fourth Edition. Neurosurgery.

[14] Wintermark M., Sanelli P.C., Anzai Y., Tsiouris A.J., Whitlow C.T. (2015). Imaging evidence and recommendations for traumatic brain injury: Advanced neuro- and neurovascular imaging techniques. Am. J. Neurorad..

[15] Yuh E.L., Cooper S.R., Ferguson A.R., Manley G.T. (2012). Quantitative CT improves outcome prediction in acute traumatic brain injury. J. Neurotrauma..

